# Macrolide Resistance in *Mycoplasma pneumoniae*, Israel, 2010

**DOI:** 10.3201/eid1706.101558

**Published:** 2011-06

**Authors:** Diana Averbuch, Carlos Hidalgo-Grass, Allon E. Moses, Dan Engelhard, Ran Nir-Paz

**Affiliations:** Author affiliation: Hadassah–Hebrew University Medical Center, Jerusalem, Israel

**Keywords:** bacteria, Mycoplasma pneumoniae, macrolide resistance, Israel, emergence, epidemiology, dispatch

## Abstract

Macrolide resistance in *Mycoplasma pneumoniae* is often found in Asia but is rare elsewhere. We report the emergence of macrolide-resistant *M. pneumoniae* in Israel and the in vivo evolution of such resistance during the treatment of a 6-year-old boy with pneumonia.

*Mycoplasma pneumoniae* is a leading respiratory pathogen in both pediatric (*1**,**2*) and adult (*1**,**3*) populations. Macrolides are considered the first line of therapy and are almost the only treatment for children. In recent years, alarming rates of *M. pneumoniae* with macrolide resistance (<90%) have occurred in eastern Asia, including the People’s Republic of China, Japan, and Korea (*2**,**4**–**7*). This was initially reported in children; however, a surge of resistance in adults was recently reported (*2**,**4**,**7*). Macrolide-resistant *M. pneumoniae* has also been suggested to be associated with a longer course of disease (*2**,**4*).

In the Western Hemisphere, lower rates of macrolide resistance have been reported (<10%), however, several epidemics with notable complications have occurred (*8**–**11*). We report the detection of macrolide resistance in *M. pneumoniae* in Israel.

## The Study

A previously healthy 6-year-old boy was hospitalized after 2 weeks with fever up to 40°C. At onset of illness, a diagnosis of pharyngitis was made. *Streptococcus pyogenes* was isolated from his throat, and amoxicillin was prescribed without any clinical response. Later, a clinical diagnosis of sinusitis was made, and amoxicillin-clavulanate was prescribed. A chest radiograph done at that time reportedly showed no abnormalities. Laboratory investigation before admission showed leukocytosis of 19,600 cells/mm^3^ with 2,200 monocytes/mm^3^ and 7,600 neutrophils/mm^3^; L-lactate dehydrogenase (LDH) was 1,854 U/L (reference value up to 600 U/L).

Ten days after the beginning of his illness, his fever decreased for 2 days and then reappeared, together with cough, resulting in hospitalization. At admission, pneumonia of the right middle and lower lobe was confirmed by chest radiograph. Laboratory tests showed leukocytes within normal ranges, *erythrocyte sedimentation rate* (ESR) 80/h, and C-reactive protein (CRP) 15 mg/L (reference range up to 0.5 mg/L). Treatment with penicillin was started without clinical improvement. Azithromycin (10 mg/kg/d) was added on the third day. After receiving this treatment, his leukocytes increased to 20,000 cells/mm^3^ with ESR 97/h and CRP 22.5 mg/L. The β-lactam coverage was switched to cefuroxime and later to ceftriaxone because no response was observed. Chest ultrasound showed a small pleural effusion. Bronchoscopy showed thick mucus secretions; respiratory specimens tested were negative for respiratory syncytial virus, influenza viruses A and B, parainfluenza virus, human metapneumovirus, and adenovirus, as were results of urine tests for *Legionella* spp. and blood tests for pneumococcal antigen and cryptococcal antigen.

Throat swab specimens were collected and DNA extracted by boiling. Samples were positive for *M. pneumoniae* by real-time PCR based on the detection of a 188-nt amplicon from the 3′ region of the repeat RepMP4 (primers: MpP1–1217_F-GTTGAAGAACGCCCAAGTGAA; MpP1–1292_R-CCGGTGGTTTGGAGCAAA). The target DNA was amplified by using Polymerase (SYBR Green) master mix (KapaTM SYBR Fast qPCR; Kapa Biosystems, Cape Town, South Africa), followed by a melt curve analysis (Rotor-Gene 6000; Corbett Research, Mortlake, NSW, Australia) with 1:30 polymerase activation at 95°C, followed by 42 cycles of 95°C for 10 s, 60°C for 18 s, 72°C for 8 s. The assay has an analytical sensitivity of 10 fg DNA or 1 to 3.3 genome copies.

**Figure 1:**
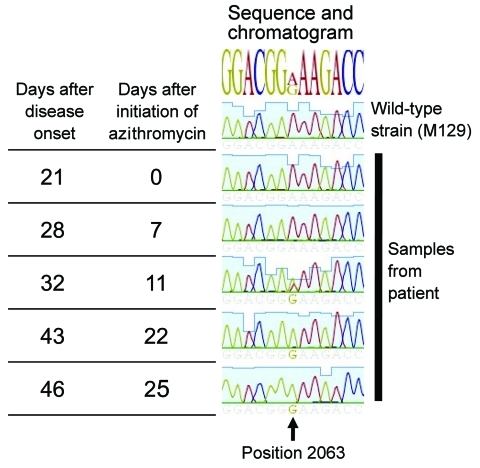
Time elapsed and chromatogram of wild-type *Mycoplasma pneumonia* strain M129 (ATCC 29342) compared with results from 5 samples from a 6-year-old boy in Israel. The A2063G mutation is shown to be evolving during treatment and predominates at the end.

Despite the *M. pneumoniae*–positive throat swab at admission, no clinical response was observed for treatment with azithromycin for 8 days. This led to the suspicion of macrolide-resistant *M. pneumoniae*. Samples were further analyzed for possible resistance-associated mutations (A2063G/C, A2064G, and A2067G) that constitute 98% of macrolide resistance–associated mutations in *M. pneumoniae* (*2*). Real-time PCR of domain V region of the 23S rRNA gene with a high-resolution melt curve analysis utilizing the SYBR Green amplification primers was used (*10*). The emergence of resistant mutants during treatment was confirmed by sequence analysis of the whole region with additional PCR of a larger amplicon size (*10*) (Figure 1). This confirmed the presence of the resistant A2063G mutation on the later samples.

Accordingly, on hospital day 21, treatment was switched to doxycycline, 4 mg/kg 2×/d for 5 days, with a prompt response; the fever subsided, the patient felt well, and the child was discharged. However, on day 33 from his first admission, he was readmitted with fever, cough, and hypoxia (oxygen saturation 85%). Chest radiograph showed atelectasis in the right middle lobe and lingula. PCR of a throat swab specimen for *M. pneumoniae* was again positive. Treatment with ciprofloxacin was started with prompt response; hypoxia subsided gradually and fever normalized. The child was treated with ciprofloxacin for 4 weeks and fully recovered.

**Figure 2:**
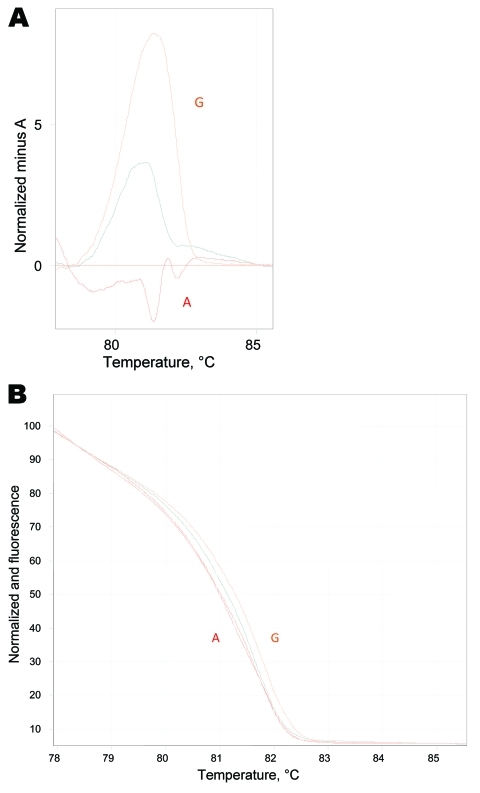
Real-time PCR high-resolution melting assay. A) High-resolution melt profiles are shown for wild-type (WT) *Mycoplasma pneumoniae* A (A2063A), macrolide resistance mutation G (A2063G) sample, and the mixed genotype sample in the normalized graph mode. B) Temperature-shifted difference graph demonstrates the deviations between WT, resistant, and mixed samples. The WT isolate has been selected to normalize the temperature shift graph and displays the deviation of samples from it.

During January 2010–August 2010, we observed a surge in *M. pneumoniae*–associated respiratory disease (30 cases during 8 months compared with 2 cases during 2009) at the Hadassah–Hebrew University Medical Centers. The Centers comprise 2 hospitals with a total of 1,000 beds in the city of Jerusalem. The Ein-Kerem campus is a tertiary center serving west Jerusalem, while the Mount Scopus campus is a secondary hospital providing primary care services to east Jerusalem. Samples from both hospitals are processed at Ein-Kerem’s clinical microbiology laboratory. During the survey period, 274 samples were submitted, of which 42 samples from 30 patients were positive for *M. pneumoniae*. Following this case, we screened all *M. pneumoniae*–positive samples for macrolide resistance*.* In 9 of 30 patients tested, we found the A2063G mutation, which was confirmed by sequencing. In 2 pediatric patients, a mixed population of both resistant (A2063G) and sensitive (A2063A) organisms was identified (Figure 2). Notably, a 31-year-old woman was found to carry the A2063G mutation as well. No significant difference was found in clinical parameters between the 9 patients with resistant and 19 patients with sensitive *M. pneumoniae* species (Table). Other than our propositus case, we did not observe in our sample a longer course of disease in patients with macrolide-resistant *M. pneumoniae*, as suggested in previous studies (*6**,**12*).

**Table:** Clinical information of patients positive for *Mycoplasma pneumoniae*, according to macrolide resistance genotype, Israel, 2010

Characteristic	Macrolide-sensitive *M. pneumoniae*, n = 19*	Macrolide-resistant *M. pneumoniae*, n = 9
Mean age, y, ± SD	7 ± 14.98	9 ± 8.39
>21	3	1
Admission, mean d, ± SD	4 ± 6.6	3 ± 8.2
Pneumonia confirmed by chest radiograph	16	9
Male/female	6/13	4/5
Ethnicity (Arab/Jewish)	5/14	1/8
Complications†	3	0
Concurrent conditions‡	4	2
Prior antibiotic use	9	6
Prior macrolide use	3	2

*Values are no. patients except as indicated. Clinical data were not available for 2 patients. †Two patients had Stevens-Johnson syndrome, and Still’s disease developed in 1 patient. ‡Four patients had asthma, 1 had metabolic syndrome with cardiomyopathy, and 1 had carcinoma of the pancreas.

## Conclusions

The observed rate of resistance in our hospital-based patients in the current surge of *M. pneumoniae*–associated cases is 30%. A2063G was the only resistance-associated mutation we found out of 4 possible mutations the assay we used can find. This is not surprising because this mutation is reportedly responsible for 90.5% of resistant *M. pneumoniae* (*2*). As recently reported from Japan, China, and Germany (*2**,**4**,**7*), we also found resistance in an adult patient. The simultaneous finding of both the macrolide-resistant (A2063G) and the macrolide-sensitive (A2063A) *M. pneumoniae* genotype in at least 1 sample from our propositus patient while he was receiving treatment suggests that the A2063G mutation may have evolved de novo during therapy with azithromycin. A similar phenomenon was observed recently in the closely related pathogen *M. genitalium* (*13*). Additionally, it was shown previously that exposing *M. pneumoniae* to sublethal concentrations of macrolides such as azithromycin can lead to de novo occurrence of macrolide resistance with A2063G mutations (*14*). This finding might suggest that lower concentrations of azithromycin on mucosal surfaces could lead to the induction of macrolide resistance in mycoplasmas. The phenomenon of a mixed population is unusual in *M. pneumoniae*–infected patients; analysis of the P1 gene of 102 *M. pneumoniae* isolates, including those obtained during epidemics (*15*), did not show such a phenomenon. Still, the infecting *M. pneumoniae* population may have been a mixture of both A2063A and A2063G species, which was later dominated by the resistant species. The occurrence of this phenomenon should be studied further in the community to evaluate its distribution and clarify whether it is related to the occurrence of de novo mutation or mixed infections.

Our study emphasizes again the therapeutic challenge in pediatric patients with *M. pneumoniae*–associated infections whose illlnesses do not respond to macrolide treatment. In such cases, we suggest that quinolones be considered as alternative therapy, although they are currently not approved for this indication in the pediatric populations.
